# An evaluation of the anti-angiogenic effect of the Korean medicinal formula “Sa-mi-yeon-geon-tang” in vitro and in ovo

**DOI:** 10.1186/s12906-015-0573-z

**Published:** 2015-03-05

**Authors:** Jin-Mu Yi, Ok-Sun Bang, No Soo Kim

**Affiliations:** KM-Based Herbal Drug Development Group, Korea Institute of Oriental Medicine, 305-811 Daejeon, Republic of Korea; Korean Medicine Life Science and Technology, Korea University of Science and Technology, 305-350 Daejeon, Republic of Korea

**Keywords:** Sa-mi-yeon-geon-tang, Anti-angiogenesis, Anti-cancer, Medicinal formula, HUVEC

## Abstract

**Background:**

Angiogenesis is a general hallmark of cancer; therefore, the inhibition of tumor-derived angiogenesis is considered to be an attractive target in the development of anti-cancer agents. Sa-mi-yeon-geon-tang (SMYGT), a decoction that consists of four natural medicinal products, has been traditionally prescribed in Oriental medicine to treat diverse diseases, including cancer. In the present study, we investigated the anti-angiogenic potential of SMYGT in vitro and in ovo.

**Methods:**

The anti-angiogenic potential of SMYGT was evaluated using conventional in vitro assays with human umbilical vein endothelial cells (HUVECs) and chorioallantoic membrane (CAM) assays with fertilized eggs. The expression changes of pro-angiogenic proteins and intracellular signaling in HUVECs following SMYGT treatment were determined by quantitative polymerase chain reaction, gelatinase zymography, and western blot analysis.

**Results:**

SMYGT efficiently inhibited three-dimensional capillary-like tube formation by HUVECs on extracellular matrix supports, as well as new vessel formation on CAMs. SMYGT inhibited cell adhesion to the extracellular matrix and HUVEC cell invasion through Matrigel without affecting cell proliferation, viability, and motility. These anti-angiogenic effects of SMYGT in HUVECs were related to decreases in the phosphorylation of focal adhesion kinase and the expression of matrix metallopeptidase-2 activity.

**Conclusions:**

SMYGT exhibited an anti-angiogenic potential in both in vitro and in ovo experiments, which may partially contribute to its anti-tumor effect in clinical conditions. We suggest that SMYGT may be a promising source material for the development of anti-cancer chemotherapeutics that target angiogenesis.

**Electronic supplementary material:**

The online version of this article (doi:10.1186/s12906-015-0573-z) contains supplementary material, which is available to authorized users.

## Background

Because cancer is a complex, multi-factorial disease, treating cancer based on a single-target/single-component strategy may be less effective than using a multi-target/multi-component strategy. In fact, oncology department currently administer various combination of chemical and/or recombinant drugs to cancer patients, and many ongoing clinical studies are investigating the efficacy of cocktail therapies in different types of cancer [1-4]. Medicinal herbs or the prescription drugs derived from them, which have been traditionally used for disease management, can be promising source materials in the development of anticancer drugs because they are originally based on multi-target/multi-component strategies. Additionally, medicinal herb extracts can be prescribed as adjuvants to produce synergistic effects with conventional anticancer drugs or to relieve the adverse side effects of the anticancer drugs. The rationale of using a combination of medicinal plants was demonstrated by Cheng’s research group, who showed that the four-herb Chinese medicine PHY906 reduced CPT-11 (irinotecan)-induced intestinal toxicity and increased the anticancer activity of CPT-11 through multiple mechanisms that act simultaneously [5]. Natural products, particularly botanical drugs, have been shown to be effective in cancer prevention or treatment on their own or in combination with chemotherapeutics [6-8].

In traditional Oriental medicine, cancer is referred to as an accumulation of mass in the body. Therefore, natural products that aim to soften and resolve the hard mass or lumps have long been used to treat cancer. Sa-mi-yeon-geon-tang (SMYGT) is a decoction composed of the combination of four natural products with medicinal values, including Sargassum, Laminariae Thallus, Prunellae Spica, and Ostreae Concha (Table 1) in an equal weight ratio. SMYGT and its component drugs have been used to soften and dissipate the abnormal hard mass [9]. The name of the formula translates as follows: Sa (four)-mi (taste)-yeon (soften)-geon (hardness)-tang (decoction). In 1983, a Chinese research group reported that SMYGT showed anti-tumor effects when administered to 511 cancer patients, including liver, stomach, and colorectal cancer [10]. Bae et al. demonstrated that modified SMYGT which included three additional herbs, showed anti-cancer or anti-metastatic effects by inhibiting DNA topoisomerase I activity and angiogenic events [9]. When administered to experimental mice with 1,2-dimethylhydrazine-induced murine colorectal cancer, modified SMYGT that included four additional herbs demonstrated anti-carcinogenic activity. However, the anticancer mechanism of SMYGT itself is unknown. In our preliminary in vitro activity screening, SMYGT was identified as a hit that showed anti-angiogenic potential. In this study, we demonstrate that SMYGT has anti-angiogenic potential in vitro and in ovo, and we suggest a feasible underlying mechanism.

**Table 1:** **Four components of SMYGT**

**Herbal name**	**Latin name**	**Genus**	**Weight Ratio**
Sargassum	*Sargassum fusiforme*	Sargassaceae	1
Laminariae Thallus	*Laminaria japonica*	Laminariaceae	1
Prunellae Spica	*Prunella vulgaris*	Labiatae	1
Ostreae Concha	*Ostrea gigas*	Ostreidae	1

## Methods

### Preparation of SMYGT extracts

The four components of SMYGT were purchased from Kwangmyungdang Medicinal Herbs (Ulsan, Republic of Korea). Their identities were confirmed by Dr. Go Ya Choi, Herbal Medicines Resources Group, Korea Institute of Oriental Medicine (KIOM). The voucher specimens of Sargassum (KIOM010011), Laminariae Thallus (KIOM010019), Prunellae Spica (KIOM010023), and Ostreae Concha (KIOM010022) were deposited at KM-Based Herbal Drug Development Group, KIOM. The crude water extract was prepared by boiling a 300 g of finely pulverized dried materials that consist of equal ratio of each component (75 g of each component) in 8 L of distilled water for 4 h. The extract was filtered through Whatman No.2 filter paper, concentrated in a rotary evaporator (EYELA, Tokyo, Japan), and lyophilized in a freeze dryer (Ilshin Bio Base, Dongducheon, Republic of Korea) The yield of the extraction (78.5 g) was approximately 26.2% (w/w). The extract was dissolved in phosphate-buffered saline (PBS) at a concentration of 80 mg/mL and stored at −70°C until use.

### Cell culture

Human umbilical vein endothelial cells (HUVECs) were purchased from Lonza (Walkersville, MD, USA), and cultured with an EGM-2 endothelial cell growth media kit (Lonza) in a humidified atmosphere with 5% CO_2_ at 37°C. The culture medium was replaced every other day. All in vitro experiments were performed using HUVECs at a passage number less than 10.

### Cell proliferation and viability

One day before drug treatment, 50,000 cells per well were inoculated into a 24-well tissue culture plate containing 475 *μ*L of EGM-2. Cells were treated with 25 *μ*L of serially diluted test drugs and were maintained for various periods. Sulforaphane (5 *μ*M) and vehicle (PBS) were administered in parallel as positive and negative control drugs, respectively. To determine cell proliferation and viability, the cultured medium was saved, and the cells were washed with PBS and trypsinized. The cells were resuspended in the saved cultured medium. The numbers of total (viable and dead) and dead cells were determined using an ADAM-MC automatic cell counter (NanoEnTek, Seoul, Republic of Korea) as described by Yi et al. [11]. In brief, the number of total (viable and dead) cells was determined using the AccuStain T solution, a cell lysis solution supplemented with a cell membrane-impermeable fluorescent dye (propidium iodide, PI). The number of dead cells was determined using the AccuStain N solution, a normal saline solution containing PI. The 1:1 mixtures of cell suspension with AccuStain T or with AccuStain N were loaded into the T and N channels of AccuChip, respectively. The number of total cells and the cell viability were automatically calculated by the following equation: cell viability (%) = [No. of AccuStain T positive total cells – No. of AccuStain N positive dead cells]/[No. of AccuStain T positive total cells] × 100.

### In vitro tube formation assay

The effect of SMYGT on HUVEC tube formation was evaluated using the Cultrex In Vitro Angiogenesis Assay Kit (Trevigen, Gaithersburg, MD, USA), according to the manufacturer’s instructions. Briefly, HUVECs were resuspended in EGM-2 containing SMYGT (100 ~ 400 *μ*g/mL) or vehicle (PBS) and then seeded at 1.5 × 10^4^ cells/well of 96‐well plates pre-coated with basement membrane extracts (BME). Sulforaphane (5 *μ*M) was administered in parallel as a positive control drug. After a 12 h incubation period, images of the capillary-like tube networks were obtained using an inverted microscope. The quantification of tube length and branch points was performed with MetaMorph image analysis software (Molecular Devices, Downingtown, PA, USA).

### Chick chorioallantoic membrane (CAM) assay

The anti-angiogenic potential of SMYGT was evaluated with an in ovo CAM assay. Fertilized eggs purchased from CJ (Seoul, Republic of Korea) were incubated at 37°C in an egg incubator (R-COM, Gimhae, Republic of Korea), with 70% relative humidity. This time point was designated as embryonic day (ED) 0. At ED 3, approximately 2 ~ 3 mL of albumin was removed, and an eggshell window was constructed. At ED 4.5, the test drugs dissolved in 80% ethanol were loaded on a one-quarter sized Thermanox coverslip (Nunc, Naperville, IL, USA) and the solvent was completely evaporated in a clean bench. The dried Thermanox coverslip containing drugs were then applied to the CAM. Solvent only and 1 *μ*g/egg of retinoic acid (RA, Sigma-Aldrich, St. Louis, MO, USA) was loaded in parallel as a negative and a positive control, respectively. At ED 6.5, the inhibition zone of angiogenesis was visualized by injecting 10% skimmed milk into the CAM. The drug response was scored as positive when CAM showed an avascular zone that was similar to the RA-treated CAM.

### Wound healing assay

HUVECs were cultured in 24-well plates to > 90% confluence. Using a 200 *μ*L pipet tip, a straight scratch was made through the cell sheet. The cells were photographed (t = 0 h), rinsed with fresh growth medium and then further incubated in fresh growth medium containing SMYGT or a vehicle (PBS). Sulforaphane (5 *μ*M) was administered in parallel as a positive control drug. After 12 h of incubation, digital images were recorded using an inverted microscope, and quantification of the healed area (%) was determined using the MetaMorph image analysis software according to the following formula: healed area (%) = [1-wounded area (t = 12 h)/wounded area (t = 0 h)] × 100.

### Cell adhesion assay

HUVECs (2 × 10^4^) were inoculated into 24-well plates pre-coated with Matrigel (BD, NJ, USA) in a growth medium containing SMYGT or a vehicle (PBS). Sulforaphane (5 *μ*M) was administered in parallel as a positive control drug. After 2 h, wells were washed with PBS to remove unattached cells. The adherent cells were fixed with 70% ethanol for 10 min and visualized with a commercially available cell staining solution (Trevigen). The cells were washed with water and completely dried by turning the plates upside down. Stained cells in three randomly selected fields per well (× 100 magnification) were digitally captured and counted using an inverted microscope.

### Cell invasion assay

The invasion assays were performed in 24-well plates equipped with cell culture inserts with a transparent PET membrane containing 8 *μ*m pores (BD). One day before the assay, HUVECs were serum-starved in EBM basal medium (Lonza) supplemented with 0.5% FBS. The cells were harvested and resuspended at a concentration of 5 × 10^5^/mL in serum-free medium. Two hundred microliters (1×10^5^ cells) of resuspended cell solution with SMYGT or vehicle (PBS) were added to the inserts pre-coated with diluted Matrigel. EGM-2 complete medium was added to the bottom chamber as a chemo-attractant. Sulforaphane (5 *μ*M) was administered in parallel as a positive control drug. After 16 h, the media were carefully aspirated from the inserts and the membrane filters were fixed with 70% ethanol for 10 min. The cells on the upper surface of the filters were completely removed using cotton swabs, and the invading cells on the opposite surface of the filter were stained with methylene blue. The GAPDH-visualized invading cells in three randomly selected fields per well (× 100 magnification) were digitally captured and counted using an inverted microscope.

### Activity gel of matrix metallopeptidase-2 (MMP2)

The activity of MMP2 secreted by HUVECs into the culture media was determined using an MMP activity gel. The culture media containing 40 *μ*g of protein were loaded onto a 7.5% sodium dodecyl sulphate -polyacrylamide gel electrophoresis (SDS-PAGE) gel that had been copolymerized with 1 mg/mL of gelatin (Sigma-Aldrich). After washing the gels 4 times to remove SDS, the enzyme activity was initiated by incubating the gel in a substrate buffer containing 50 mM Tris (pH 7.6), 1 *μ*M ZnCl_2_, and 5 mM CaCl_2_ overnight at 37°C. The degradation of the gelatin substrate by MMP2 was visualized by staining the gel with a 0.1% Coomassie blue solution.

### Quantitative polymerase-chain-reaction (qPCR)

The total RNA was extracted from HUVECs with an Easy-spin™ Total RNA Extraction Kit (iNtRON Biotechnology, Seoul, Republic of Korea). Complementary DNAs (cDNAs) were synthesized using the iScript™ cDNA Synthesis Kit (Bio-Rad, Hercules, CA, USA), according to the manufacturer’s instructions. qPCR, which was performed with SsoAdvanced™ Universal SYBRR Green Supermix (Bio-Rad) on a CFX™ 96 Real-Time Detection System (Bio-Rad), was initiated by activation at 95°C for 30 s, followed by 40 cycles of amplification (denaturation at 95°C for 5 s, annealing/elongation at 60°C for 30 s). Gene expression was normalized to the housekeeping gene for glyceraldehyde-3-phosphate dehydrogenase (GAPDH). Each sample was measured in triplicates. The sequences of gene specific primers and their amplicons are illustrated in Table 2.

**Table 2:** **Sequences of gene specific primers**

**Target**	**Forward (5′ → 3′)**	**Reverse (5′ → 3′)**	**Amplicon (bp)**
MMP2	ATGCCGCCTTTAACTGGAG	GGGAAGCCAGGATCCATTTT	103
GAPDH	AAGGCTGAGAACGGGAAG	GGACTCCACGACGTACTC	114

### Western blot analysis

Western blot analysis was performed as previously described [11]. The total focal adhesion kinase (FAK) and phospho-FAK (pFAK, Y397) antibodies were obtained from Cell Signaling Technology (Danvers, MA, USA), and the β-actin antibody was obtained from Bio-Rad.

### Statistics

Statistical data analysis was performed using one-way analysis of variance and the level of statistical significance was set at *p* < 0.05.

## Results

### SMYGT inhibits angiogenesis in vitro and in ovo

**Figure 1:**
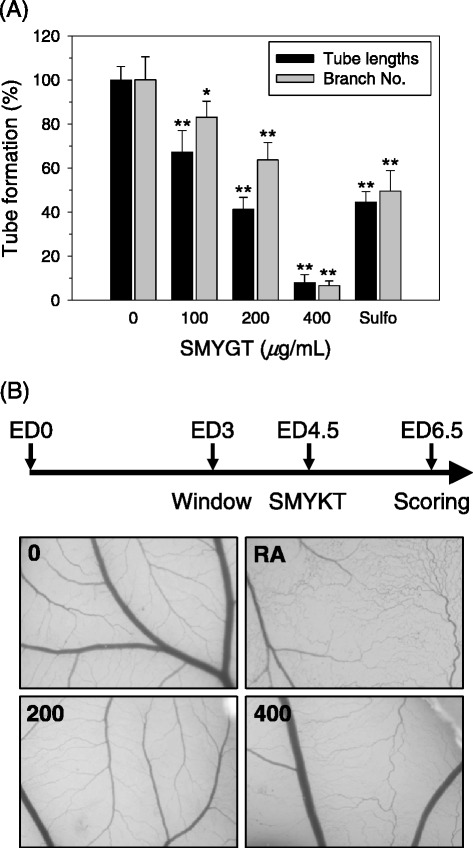
**Anti-angiogenic potential of SMYGT in vitro and in ovo. (A)** HUVECs cultured on BME-pre-coated supports were exposed to different concentrations of SMYGT for 12 h. The tube length and branch numbers were determined by analyzing digitally captured images. Sulforaphane (Sulfo, 5 *μ*M) was administered in parallel as a positive control drug. Relative tube formation was determined by comparing each group with the vehicle treated group (0 *μ*g/mL SMYGT), and the data are presented as the means ± standard deviation (S.D.) of triplicate experiments. *P < 0.05, **P < 0.01, ***P < 0.001. **(B)** At ED4.5, A quarter size of thermanox coverslips containing variable amounts of SMYGT (0, 200, 400 *μ*g/disc) were applied to the CAMs. After 2 days of incubation, 10% skimmed milk solution was injected into the CAM for observation of the inhibition zone of angiogenesis and digital images were captured. Retinoic acid (RA, 1 *μ*g/disc) used as a control for inhibition of new vessel formation.

The anti-angiogenic potential of SMYGT was evaluated with an in vitro tube formation assay mediated by HUVECs. In the absence of SMYGT, HUVECs constructed blood vessel-like tubes by connecting with neighboring cells (Additional file 1A, 0 *μ*g/mL of SMYGT). However, the SMYGT treatment interrupted this intercellular connection by inhibiting tube formation (Additional file 1A, 100–400 *μ*g/mL of SMYGT). Image analysis revealed that the degree of HUVEC-mediated angiogenesis, based on tube length and branch numbers, was decreased by SMYGT in a dose-dependent manner (Figure 1A). The inhibitory effect of 200 *μ*g/mL of SMYGT was comparable to that of 5 *μ*M of sulforaphane, a positive control drug, and tube formation was completely inhibited by 400 *μ*g/mL of SMYGT.

Next, we confirmed the anti-angiogenic potential of SMYGT in ovo. Fertilized eggs were treated with SMYGT (0-400 *μ*g/egg) on ED4.5, and the degree of vessel formation on CAMs was scored 2 days later. Blood vessel formation was impeded, and the avascular zone was increased by SMYGT in a dose-dependent manner (Figure 1B). Retinoic acid (RA, 1*μ*g/egg) was used as a positive control. The proportion of eggs exhibiting anti-angiogenic effects is summarized in Table 3. Dead embryo was not observed in the tested dose ranges of SMYGT, indicating that SMYGT-mediated anti-angiogenesis in ovo was not due to SMYGT toxicity.

**Table 3:** **Summary of anti-angiogenic effect of SMYGT in chick CAM assays**

**Drugs**	**Dose (** ***μ*** **g per egg)**	**Tested eggs (n)**	**Eggs showing anti-angiogenesis (n (%))** ^*^
Vehicle	-	10	1 (10.0)
RA	1	10	9 (90.0)
SMYGT	200	10	3 (25.0)
	400	10	8 (66.6)

^*^CAM showing an avascular zone similar to RA-treated CAM was scored as positive.

### SMYGT potently inhibits cell adhesion and the invasiveness of HUVECs

**Figure 2:**
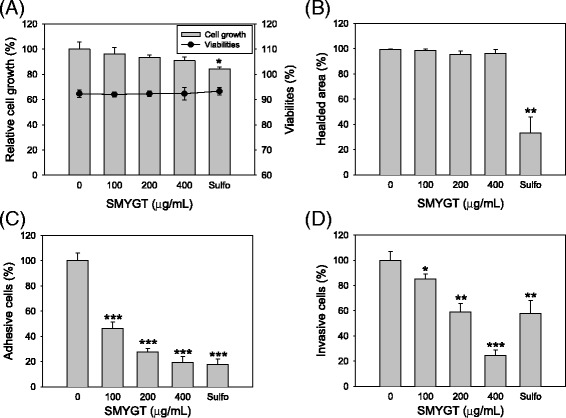
**Effects of SMYGT on the HUVEC-mediated angiogenic process in vitro. (A)** HUVECs were cultured in the presence and absence of SMYGT for 24 h and measured using an automatic cell counter to determine cell growth and viability. **(B)** Scratches were applied to the lawn of HUVECs, and then cells were cultured in the presence or absence of SMYGT. After 12 h, the relative cell motility was determined by analyzing digital images. **(C)** HUVECs were resuspended in culture medium containing various concentrations of SMYGT and then inoculated in culture plates that were pre-coated with Matrigel. After 2 h, the adherent cells were fixed, stained, and then counted under a microscope. **(D)** Serum-starved HUVECs were inoculated into the cell culture inserts pre-coated with Matrigel and then cultured in the presence or absence of SMYGT. After 16 h, chemotactic HUVECs that invaded through the Matrigel were stained and counted under a microscope. Sulforaphane (Sulfo) was included in these assays for a positive control drug. All data, except viability, are presented as the relative means ± S.D. of triplicate experiments compared to the vehicle treatment group. *P < 0.05, **P < 0.01, ***P < 0.001.

To understand the anti-angiogenic activity of SMYGT, the cellular responses of HUVECs to SMYGT treatment were investigated in vitro. First, we determined the cytotoxicity of SMYGT in HUVECs based on cellular membrane integrity. When HUVECs were exposed to increasing concentrations of SMYGT, no significant morphological changes was observed (Additional file 1B), and HUVECs maintained normal cell growth and a viability of > 90% in concentrations up to 400 *μ*g/mL of SMYGT (Figure 2A). These data suggest that the anti-angiogenic potential of SMYGT, as demonstrated by HUVEC-mediated tube formation and in ovo CAM assays, was not due to its cytotoxicity. An in vitro wound healing assay used to determine cell mobility demonstrated that although a slight inhibitory effect was observed at SMYGT concentrations ≥200 *μ*g/mL, but HUVECs showed normal cell movement when compared to cells treated with 5 *μ*M of sulforaphane, a positive control drug (Additional file 1C). Image analysis did not reveal that SMYGT induced any significant changes in cell movement (Figure 2B). In contrast, SMYGT reduced endothelial cell adhesion to the extracellular matrix (Additional file 1D), and image analysis revealed that 400 *μ*g/mL of SMYGT reduced cell adhesion by 80.6% (Figure 2C). Furthermore, SMYGT reduced cellular invasiveness through the membrane (Additional file 1E), and the inhibitory effect of 200 *μ*g/mL of SMYGT was comparable to that of 5 *μ*M of sulforaphane (Figure 2D).

### SMYGT down-regulates angiogenesis-related FAK signaling and MMP2 expression

**Figure 3:**
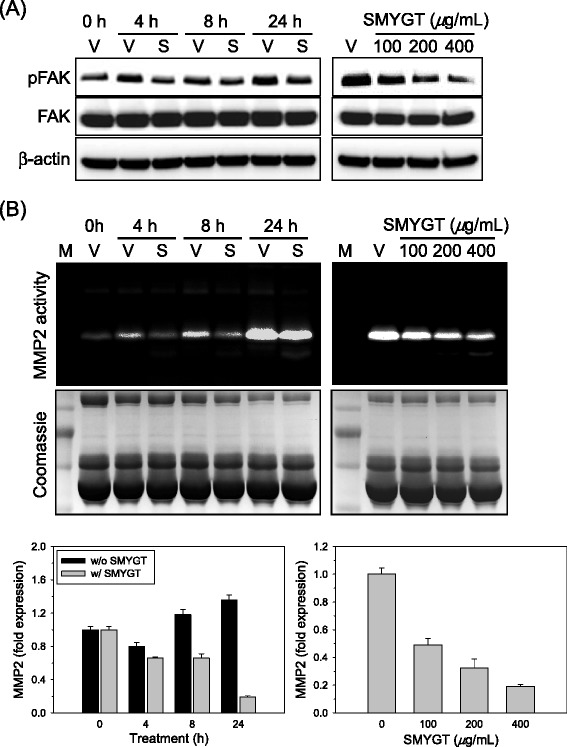
**The effects of SMYGT on FAK signaling and MMP2 expression in HUVECs. (A)** Time-(left, 200 *μ*g/mL of SMYGT) and dose-(right, 24 h) dependent effect of SMYGT on phosphorylation of the intracellular FAK protein were determined using western blot analyses. β-actin was used to confirm equal loading of total proteins. **(B)** Time-(left, 200 *μ*g/mL of SMYGT) and dose-(right, 24 h) dependent changes of MMP2 enzyme activities and mRNA induced by SMYGT were determined by zymography (top) and qPCR (bottom), respectively. A PAGE gel stained with Coomassie blue solution (middle) is presented to demonstrate equal loading of the proteins for the MMP2 zymography. V, vehicle; S, SMYGT.

As shown in Figure 2C, SMYGT potently inhibited the adhesion of HUVECs to matrix supports. Because integrin-FAK signaling plays a key role in cell adhesion, and activation of integrin leads to phosphorylation of FAK [12], we determined the level of phosphorylated FAK following SMYGT treatment in HUVECs. SMYGT reduced the phosphorylation of FAK compared to vehicle treatment (Figure 3A). The total FAK and β-actin, a loading control for western blot analysis, were not changed by SMYGT treatment. Therefore, SMYGT may affect cell adhesion by down-regulating integrin-FAK signaling pathway.

Invasive cell migration is an important function of both endothelial and cancer cells during angiogenesis. Cellular invasiveness depends on the enzymatic degradation of the extracellular matrix, which is mediated by MMPs. We determined the effect of SMYGT on MMP2 and 9 enzyme activities and intracellular mRNA by gelatinase activity gels and qPCR, respectively. The activity gel assay demonstrated that SMYGT inhibited MMP2 enzyme activity in a time-(upper left) and dose-(upper right) dependent manner. MMP9 enzyme activity was too low to be detected in the activity gel assay. The decrease in MMP2 enzyme activity triggered by SMYGT was observed in parallel with a decrease in intracellular MMP2 mRNA, as determined by qPCR (Figure 3B, lower panel); therefore, SMYGT may inhibit HUVEC invasiveness by down-regulating MMP2 activity at the transcription level.

## Discussion

Angiogenesis is a multi-stepped process of de novo vessel formation from the established blood vasculature by endothelial cells. It is essential for continuous tumor growth because it supplies tumors with nutrients and oxygen and removes cellular waste, which may be toxic to cancer cells, from the tumor mass. Since the idea of tumor angiogenesis was suggested in the 1970s by Folkman [13], the blockade of tumor-induced angiogenesis has been considered to be an attractive anticancer strategy because anti-angiogenic agents can be used irrespective of cancer types because tumor-induced angiogenesis is a general hallmark of cancer, and the agents target vascular endothelial cells with genomic stability, which rarely develop drug resistance [14,15]. Angiogenesis is a process that is coordinated by the functional balance between pro-angiogenic and anti-angiogenic factors. Therefore, anti-angiogenesis can be accomplished by blocking the signaling pathway mediated by pro-angiogenic proteins, such as vascular endothelial growth factor (VEGF) and basic fibroblast growth factor, and/or by up-regulating the expression of endogenous anti-angiogenic proteins, such as endostatin and tissue inhibitor of metalloproteinases-1 (TIMP-1).

In this study, we described the novel anti-angiogenic effect of SMYGT, an extract that is a traditional Korean medicinal cocktail. We demonstrated that SMYGT exerts its anti-angiogenic potential in vitro by inhibiting the cell adhesion and invasiveness of endothelial cells. We also demonstrated that SMYGT inhibits intracellular FAK signaling by modulating its phosphorylation status and down-regulates MMP2 activity by reducing intracellular levels of MMP2 mRNA. In general, phosphorylation of FAK is known to be related to the adhesion of cells to the extracellular matrix, as well as the motility and survival of endothelial cells [16]. We observed that SMYGT down-regulated FAK signaling and inhibited endothelial cell adhesion to the extracellular matrix, but cell proliferation, viability, and mobility were not affected by SMYGT. This differential response of HUVECs to SMYGT may be related to the partial inhibition of FAK by SMYGT, which is adequate to affect cell adhesion but not sufficient to affect cell survival, proliferation, and motility. FAK signaling is considered to be a promising anti-cancer target because phosphorylation of FAK, particularly at Y397, is also critical for angiogenesis, metastasis, and the invasion of tumor cells because of its effects on regulating cell adhesion to the extracellular matrix, cell survival, motility, and proliferation [17,18]. MMPs also contribute to endothelial cell-mediated angiogenesis by loosening focal cell-to-cell interaction and by degrading the basement membrane and extracellular matrix surrounding blood vessels [19,20]. In this study, we demonstrated that SMYGT could down-regulate MMP2 activity in HUVECs. However, MMP9 was not detected in a gelatinase activity gel assay using HUVEC cultured medium which is consistent with our previous studies of other anti-angiogenic medicinal herb extracts [11]. MMP2 activity has also been reported to be effectively reduced by anti-cancer agents that target tumor vessel formation [21,22]. MMP2 activity can be regulated during the transcriptional, post-transcriptional (e.g., mRNA stability), translational, and post-translation (e.g., extracellular secretion) steps. In this study, we suggest that SMYGT can down-regulate MMP2 activity by decreasing intracellular level of mRNA. However, we did not investigate whether SMYGT-mediated MMP2 regulation is controlled transcriptionally or post-transcriptionally. It is known that when endothelial cells are subjected to hypoxic conditions (low PO_2_) such as inside of a tumor mass, MMP2 expression is elevated, and its specific endogenous inhibitor TIMP-2 and the hypoxic conditions enhance MMP2-dependent endothelial cell migration [19].

SMYGT, a traditional medicinal decoction, has been used to treat cancer patients in Korean Oriental medicine clinics [9], and it has been revealed that this herbal prescription has an inhibitory effect on inflammation and fecal enzyme activity in an ulcerative colitis animal model [10]. Modified SMYGT has also demonstrated potentially cytotoxic activity against various cancer cells, such as A549, SK-OV-3, B165-F10, and SK-MEL-2, and it has been shown to suppress lung metastasis and angiogenesis in an animal model [9]. SMYGT consists of four components in equal weights. *Sargassum fusiforme* and *Laminaria japonica* are members of the brown algae family, which is widely distributed in Far East Asia, including Korea, China, and japan; *S. fusiforme* and *L. japonica* have been used as therapeutic agents for thousands of years [23,24], and polysaccharides extracted from *S. fusiforme* and *L. japonica* are known to have various biological activities, such as anti-tumor [25], anti-oxidant [24], immunity enhancing [26], and anti-hyperlipidemia activities [27]. The shell of *Ostrea gigas* has been prescribed with other medicinal herbs to treat various symptoms, such as palpitations, insomnia, dizziness, tinnitus, scrofula, subcutaneous nodules, and abdominal masses [28]. Recent studies have revealed that Ostreae Concha pharmacologically functions to boost the immune system and relieves gastric ulcers, sedation, viral infections, and tumors [29]. *Prunella vulgaris* is widely distributed in the temperate zone, and it has been used to treat inflammation, eye pain, headaches, and dizziness [30]. *P. vulgaris* has anti-inflammatory, anti-bacterial, anti-viral, and anti-tumor effects, and these pharmacological functions may be attributed to its immunomodulatory activity [31]. In the prescription, Sargassum and Laminariae Thallus primarily help to soften and resolve hard masses, Prunellae Spica reduces pathogenic heat, and Ostreae Concha alleviates pains. These ingredients enhance the efficacy of SMYGT, according to traditional oriental medicine theory. We did not determine whether the anti-angiogenic potential of SMYGT is attributed to a single component or a combination of components of SMYGY.

## Conclusion

In summary, in the present study, we demonstrated the novel anti-angiogenic activity of SMYGT, which has been traditionally used to treat diverse diseases, including cancer. SMYGT exerts its anti-angiogenic potential by down-regulating the pro-angiogenic protein MMP2 and by inhibiting the cell adhesion-related FAK signaling pathway. Further rigorous studies should be performed to determine the detailed anti-angiogenic mechanism of SMYGT and to identify its active constituents to develop anti-cancer drugs using SMYGT or its active constituents.

## Additional file

Additional file 1:
**Determination of anti-angiogenic potential of SMYGT in vitro.** To investigate anti-angiogenic potential of SMYGT, HUVEC was exposed to SMYGT (0-400 *μ*g/mL). PBS and sulforaphane (Sulfo, 5 *μ*M) were treated as a vehicle and a positive control, respectively. Cells were observed under the inverted microscope at indicated magnifications. (A) tube formation (× 40), (B) cell growth (× 200), (C) cell mobility (× 100), (D) cell adhesion (× 100), and (E) cell invasion (× 100). At least three independent experiments were performed and representative images of each assay are shown.

## Abbreviations

SMYGT: Sa-mi-yeon-geon-tang
HUVEC: Human umbilical vein endothelial cells
CAM: Chorioallantoic membrane
BME: Basement membrane extracts
ED: Embryonic day
SDS-PAGE: Sodium dodecyl sulphate-polyacrylamide gel electrophoresis
MMP2: Metallopeptidase-2
qPCR: quantitative polymerase-chain-reaction
cDNA: complementary DNA
GAPDH: Glyceraldehyde-3-phosphate dehydrogenase
FAK: Focal adhesion kinase
VEGF: Vascular endothelial growth factor
TIMP-1/2: Tissue inhibitor of metalloproteinases-1/2
