# Supplementary material for: An evaluation of the anti-angiogenic effect of the Korean medicinal formula “Sa-mi-yeon-geon-tang” in vitro and in ovo
Source: BMC Complement Altern Med. 2015 Mar 5;15:42. doi: 10.1186/s12906-015-0573-z (PMC4359561; doi:10.1186/s12906-015-0573-z)

Additional file 1. Determination of anti-angiogenic potential of SMYGT in vitro. To investigate anti-angiogenic potential of SMYGT, HUVEC was exposed to SMYGT (0-400 μg/mL). PBS and sulforaphane (Sulfo, 5 μM) were treated as a vehicle and a positive control, respectively. Cells were observed under the inverted microscope at indicated magnifications. (A) tube formation (x 40), (B) cell growth (x 200), (C) cell mobility (x 100), (D) cell adhesion (x 100), and (E) cell invasion (x 100). At least three independent experiments were performed and representative images of each assay are shown.

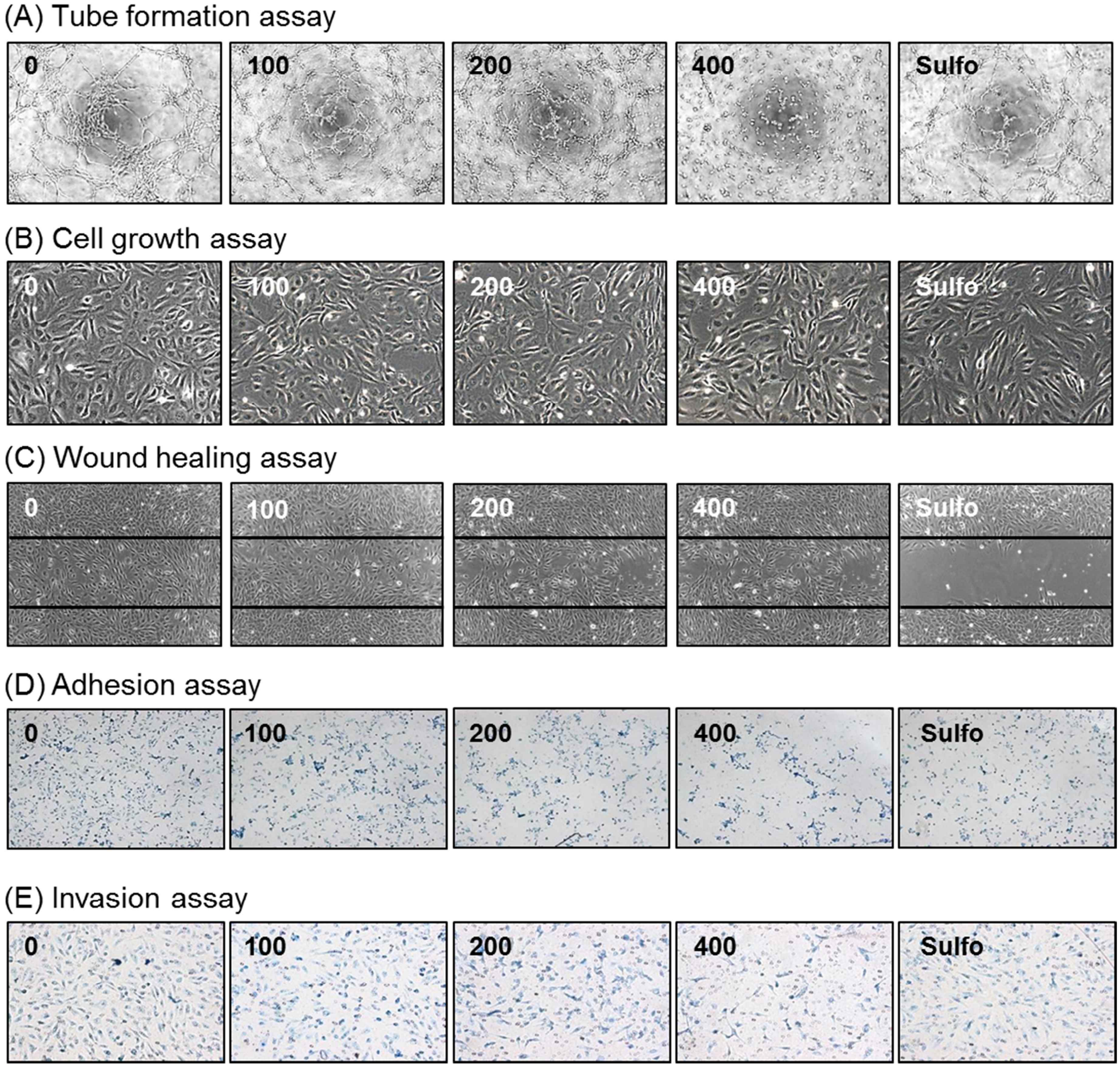

Supplement: Additional file 1: — Determination of anti-angiogenic potential of SMYGT in vitro. To investigate anti-angiogenic potential of SMYGT, HUVEC was exposed to SMYGT (0-400 μg/mL). PBS and sulforaphane (Sulfo, 5 μM) were treated as a vehicle and a positive control, respectively. Cells were observed under the inverted microscope at indicated magnifications. (A) tube formation (× 40), (B) cell growth (× 200), (C) cell mobility (× 100), (D) cell adhesion (× 100), and (E) cell invasion (× 100). At least three independent experiments were performed and representative images of each assay are shown. [file 12906_2015_573_MOESM1_ESM.pdf]
